# Rice bran wax-hexane superhydrophobic coating production: an environmental assessment

**DOI:** 10.1039/d6su00434b

**Published:** 2026-08-05

**Authors:** Maite Mañana-Garce, Brenda Resendiz-Diaz, Marco Vaccari, Monica Puccini, Colin R. Crick

**Affiliations:** a School of Engineering and Materials Science, Queen Mary University of London Mile End Road London E1 4NS UK m.mananagarce@qmul.ac.uk b.resendiz-diaz@qmul.ac.uk c.crick@qmul.ac.uk; b Department of Civil and Industrial Engineering, University of Pisa Largo Lazzarino 2 56122 Pisa Italy marco.vaccari@unipi.it monica.puccini@unipi.it

## Abstract

Food packaging waste, particularly from plastic-based materials, represents an environmental concern, accounting for nearly half of global plastic waste. While bio-based superhydrophobic coatings have recently emerged as promising alternatives, their environmental implications remain insufficiently understood. This study applies a laboratory-scale cradle-to-gate life cycle assessment (LCA) to establish the environmental profile of rice bran wax–*n*-hexane superhydrophobic coating fabrication and identify the environmental hotspots within its production stage. The analysis is based on primary laboratory-scale production data, with a functional unit representing the preparation of the coating dispersion required for coating 1 m^2^ of glass surface. Solvent selection was identified as the dominant contributor across all impact categories, accounting for at least 50% of the total impact, followed by electricity under the laboratory-scale modelling assumptions. Sensitivity analysis showed that substituting *n*-hexane with petroleum ether led to substantial reductions across all categories, with reductions exceeding 50% for eutrophication potential (64%), photochemical ozone creation potential (61%), abiotic depletion potential (elements) (53%), and terrestrial ecotoxicity potential (51%). Replacing the baseline electricity mix with renewable electricity further reduced impacts, with the most significant decrease observed in global warming potential (36%). Although petroleum ether resulted in slightly lower coating performance, it consistently achieved lower environmental impacts, highlighting the importance of integrating environmental assessment alongside functional performance during material selection. Overall, this study establishes an environmental baseline for an emerging bio-based superhydrophobic coating and demonstrates how early-stage LCA can identify fabrication hotspots and support environmentally informed process design for the development of more sustainable barrier coating technologies. Laboratory-scale LCA reveals solvent selection and associated upstream processes as the main environmental hotspots in coating dispersion preparation. Switching solvent systems and electricity supply significantly reduces environmental impacts.

## Abstract 2

Cradle-to-gate LCA of bio-based superhydrophobic coatings reveals solvent choice as the primary environmental hotspot. Strategic solvent substitution and renewable power reduce impacts by >50%, guiding sustainable barrier packaging design.

## Graphical abstract

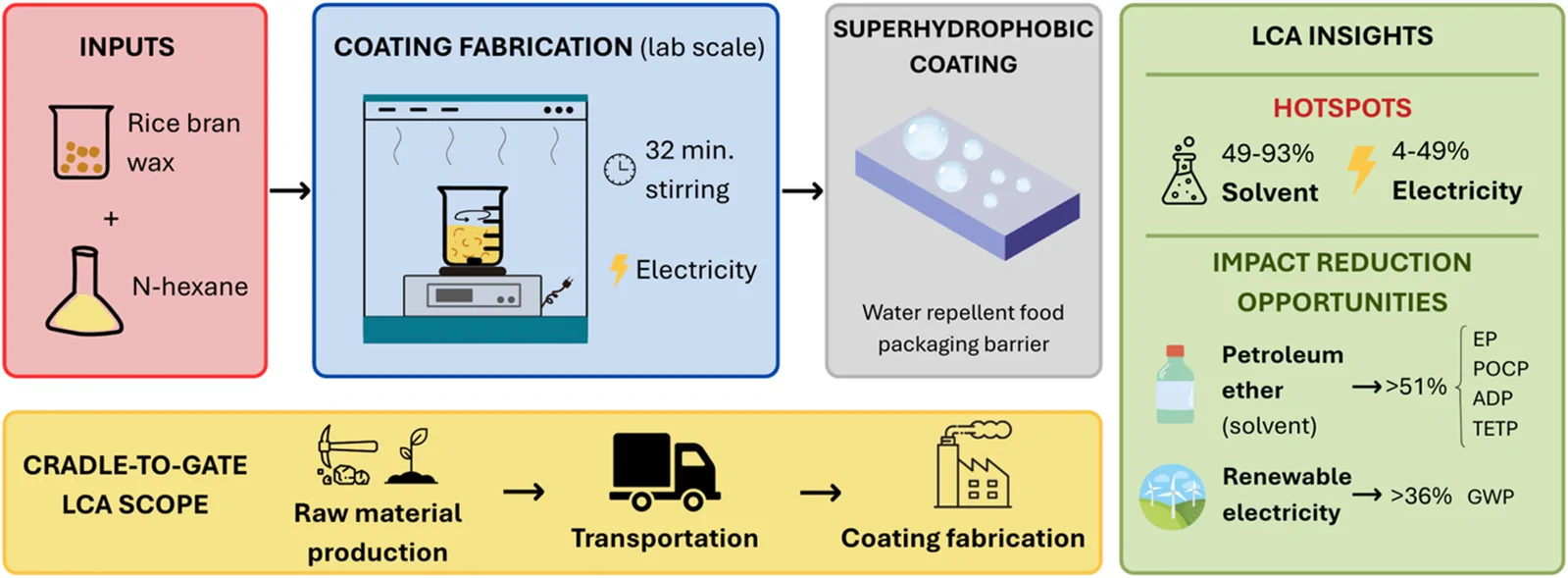

**Sustainability spotlight**

Bio-based materials are often assumed to be environmentally preferable, yet this assumption cannot replace quantitative sustainability assessment. This study combines the development of a rice bran wax-based superhydrophobic coating with an early-stage life cycle assessment to evaluate its environmental performance before scale-up. The analysis identifies the main environmental hotspots and demonstrates how prospective life cycle assessment can guide material selection, process optimization, and research priorities during technology development, reducing the risk of burden shifting. By integrating environmental assessment into the design of functional food-packaging coatings, this work advances more sustainable innovation and contributes to United Nations Sustainable Development Goal 12: Responsible Consumption and Production.

## 1. Introduction

Food packaging plays a critical role in preserving product quality and extending shelf life by acting as a crucial barrier against damage and maintaining product integrity.^1^ However, food packaging waste represents a major environmental concern, as it contributes significantly to solid waste generation,^2^ accounting for nearly half of all plastic waste generated worldwide.^3^ These materials persist in the environment for extended periods, and rely on non-renewable resources, contributing to greenhouse gas emissions, landfill accumulation, and marine pollution.^2^ Despite these drawbacks, conventional plastic materials are widely used due to their effective barrier properties, which are essential for protecting food products from external degradation.^4^

The intensifying demand for food production calls for more effective systems to manage the waste generated throughout the supply chain.^2^ Consequently, their environmental impact has driven the search for alternative packaging materials, particularly paper- and fibre-based substrates.^4^ A key limitation, however, is that these materials are hydrophilic and therefore sensitive to moisture, requiring an additional barrier layer to prevent liquid penetration.^4^ To address this limitation, surface treatments are required to enhance resistance to water and other external factors. In this context, superhydrophobic surfaces, characterized by water contact angles greater than 150° and exhibiting extreme water repellency, represent a promising strategy to achieve this.^4^ Traditionally, coatings have been applied to impart such functionality.^1^ However, this also necessitates the development of coating solutions that preserve the environmental advantages of alternative substrates rather than compromising them.^2^

Recent research demonstrates promising directions. Razavi *et al.*^5^ developed bio-based superhydrophobic coatings using naturally derived fatty acids and mineral nanoparticles, achieving advancing water contact angles exceeding 160° while demonstrating scalable fabrication and significant antibiofouling performance without relying on toxic fluorinated chemistries. Bayer,^6^ in turn, provided a comprehensive review of eco-friendly materials and fabrication processes for superhydrophobic coatings. The study highlighted that superhydrophobic coatings can be derived from natural waxes such as carnauba, beeswax, candelilla, and rice bran wax, often processed using green solvents such as water or bio-based solvents (*e.g.*, ethanol or ethyl acetate), emphasizing the role of hierarchical surface structures in achieving water contact angles above 150°. The review further noted that many existing coating formulations remain unsuitable for large-scale fabrication or prohibitively expensive for industrial implementation, highlighting the need for more robust and environmentally benign coating technologies. Lastly, Bai *et al.*^7^ offered an extensive overview of recent advances in superhydrophobic coatings across various application fields, including self-cleaning, anti-corrosion, anti-icing, oil–water separation, and biomedical applications. The review highlighted the multifunctional nature of these coatings while concluding that improving durability, wear resistance, and large-scale fabrication remains essential for their successful real-world implementation. However, as these materials move closer to practical implementation, evaluating whether their fabrication processes deliver genuine environmental benefits becomes increasingly important.

In parallel with these developments, previous studies have applied life cycle assessment to coating systems and fabrication processes, highlighting the influence of materials, solvents, and processing conditions on overall environmental burdens.^8–10^ For example, Lu *et al.*^8^ showed that electricity demand and upstream solvent production were the dominant contributors to the environmental impacts of membrane coating fabrication, highlighting that the environmental burdens associated with producing alternative green solvents can offset their perceived sustainability advantages. Similarly, Lemesle *et al.*^10^ demonstrated that simplifying coating fabrication routes can substantially reduce environmental impacts, emphasizing the importance of process design alongside material selection. Beyond fabrication processes, LCA studies on anti-fouling, anti-icing, and anti-corrosion coatings have further shown that coating technologies often involve trade-offs between functional performance, manufacturing impacts, and life-cycle benefits depending on formulation, application, and service conditions.^9,11,12^ However, despite these advances, the environmental implications of emerging bio-based superhydrophobic coatings remain largely unexplored, particularly for barrier technologies intended for food-packaging applications. This gap highlights the need for early-stage life cycle assessment to quantify the environmental impacts associated with coating fabrication and identify environmental hotspots that can guide process optimization during technology development.

Although reducing the environmental impacts associated with coating fabrication is an important component of sustainable food packaging, the overall sustainability of packaging systems depends on multiple complementary considerations beyond manufacturing. As'ad Mahpuz *et al.*^2^ describe three complementary objectives for sustainable packaging design: the use of renewable materials with appropriate end-of-life characteristics, the minimization of waste generation and environmental burdens throughout the product life cycle, and the reduction of environmental impacts associated with material production and manufacturing. The present study focuses specifically on the third objective by evaluating the environmental impacts associated with the fabrication of an emerging bio-based superhydrophobic coating through a cradle-to-gate life cycle assessment. The selected coating, previously developed by Resendiz-Diaz *et al.*,^13^ consists of a rice bran wax–*n*-hexane dispersion that was experimentally validated on fibre-based packaging substrates, demonstrating high water repellency and barrier performance for potential food-packaging applications. By isolating the manufacturing stage, the present work provides an environmental assessment of the coating dispersion preparation process within the broader framework of sustainable food packaging. Accordingly, end-of-life considerations (including recyclability, biodegradability, compostability, and the environmental performance of complete coated packaging systems) remain outside the scope of the present cradle-to-gate assessment and should be considered alongside the manufacturing-stage impacts evaluated here when assessing the overall sustainability of emerging food-packaging coatings.

The Life Cycle Assessment (LCA) method is a framework that evaluates the environmental impacts related to a material, product, or process life cycle. It enables the identification and analysis of opportunities to reduce environmental impacts.^14^ LCA has been applied to coating systems and material processing routes, including at the laboratory scale, demonstrating its suitability for assessing environmental burdens associated with formulation and fabrication processes.^8–10^ For example, comparative LCA studies have evaluated different coating fabrication routes, showing that process design and material choices can significantly influence environmental performance.^10^ Such early-stage assessments are commonly used to evaluate emerging materials prior to industrial implementation, providing insight into potential environmental trade-offs despite inherent scaling limitations.

Building on this foundation, the present work applies a laboratory-scale cradle-to-gate life cycle assessment to the aforementioned superhydrophobic coating using primary inventory data collected during fabrication. The assessment was restricted to the rice bran wax–hexane formulation previously reported by Resendiz-Diaz *et al.*,^13^ for which sufficiently detailed and reliable life cycle inventory data (detailed in Section 3) were available, ensuring methodological robustness and data consistency throughout the analysis. Because the objective was to quantify the environmental burdens associated with the preparation of the coating dispersion and identify the dominant environmental hotspots during the early stages of technology development, the LCA scope was limited to the production phase of the coating material and a cradle-to-gate system boundary was adopted. This study aims to integrate environmental assessment into the early stages of material development by establishing an environmental baseline, identifying the dominant fabrication hotspots associated with an emerging bio-based superhydrophobic coating, and providing evidence to support environmentally informed process design and the future optimization of barrier technologies for potential food-packaging applications.

The life cycle assessment was conducted in accordance with ISO 14040:2006, following the standard framework of goal and scope definition, inventory analysis, impact assessment, and interpretation.^15^

## 2. Goal and scope definition

The objective of this LCA is to (i) develop a cradle-to-gate environmental profile of rice bran wax–*n*-hexane coating dispersion preparation and (ii) identify the key environmental areas of concern (hotspots) within its production stage to guide process optimization and impact reduction.

In this study, and in line with previous literature,^11,12^ the functional unit was defined as the preparation of the quantity of rice bran wax–*n*-hexane dispersion required for coating 1 m^2^ of glass surface according to the formulation reported by Resendiz-Diaz *et al.*.^13^ Using a surface-based functional unit enhances comparability, since coating formulations can differ in the mass required to achieve equivalent surface coverage and functional performance. Glass was selected as a model substrate because its well-characterized, non-porous nature provides reproducible surface properties, including predictable absorption behavior, while enabling consistent evaluation of the dispersion preparation process independently of substrate-specific effects.^16^ The coated surface is used solely as the basis for scaling the material and energy requirements of the dispersion preparation process. Consequently, the environmental impacts reported in this study relate exclusively to the preparation of the coating dispersion and should not be interpreted as representing the environmental performance of a complete food-packaging system. The assessment does not include spray application, or the production of the coated substrate, as these processes occur outside the defined system boundary. Evaluating the environmental performance of complete coated packaging systems across their full life cycle remains the subject of future work.

The analysed system is divided into three stages: (i) raw material production (rice bran wax and *n*-hexane in particular); (ii) transportation of the materials into the facility for manufacture; and (iii) dispersion preparation, during which the wax is mechanically stirred in the solvent at room temperature (Fig. 1). This process is undertaken under fume-hood ventilation within the laboratory facilities at Queen Mary University of London, London, United Kingdom.

**Fig. 1:**
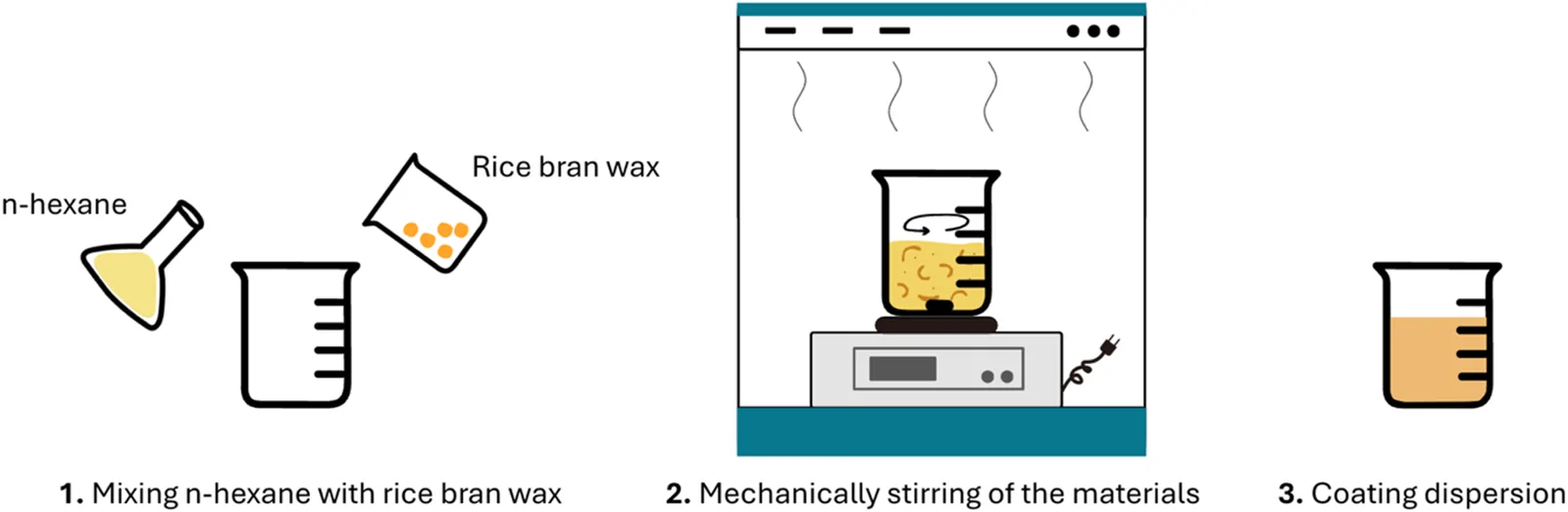
Schematic of dispersion preparation: rice bran wax dispersed in *n*-hexane *via* mechanical stirring under fume hood conditions.

The foreground output of the analysed system is the prepared rice bran wax–*n*-hexane dispersion. The system boundary terminates immediately after the dispersion preparation step. Therefore, the solvent remains contained within the prepared dispersion when it leaves the foreground system. Accordingly, spray application, solvent evaporation during drying, direct volatile organic compound (VOC) emissions, solvent recovery, and solvent waste management are excluded from the present assessment because they occur in downstream life cycle stages outside the defined system boundary. Fig. 2 illustrates the cradle-to-gate system boundary considered in this study.

**Fig. 2:**
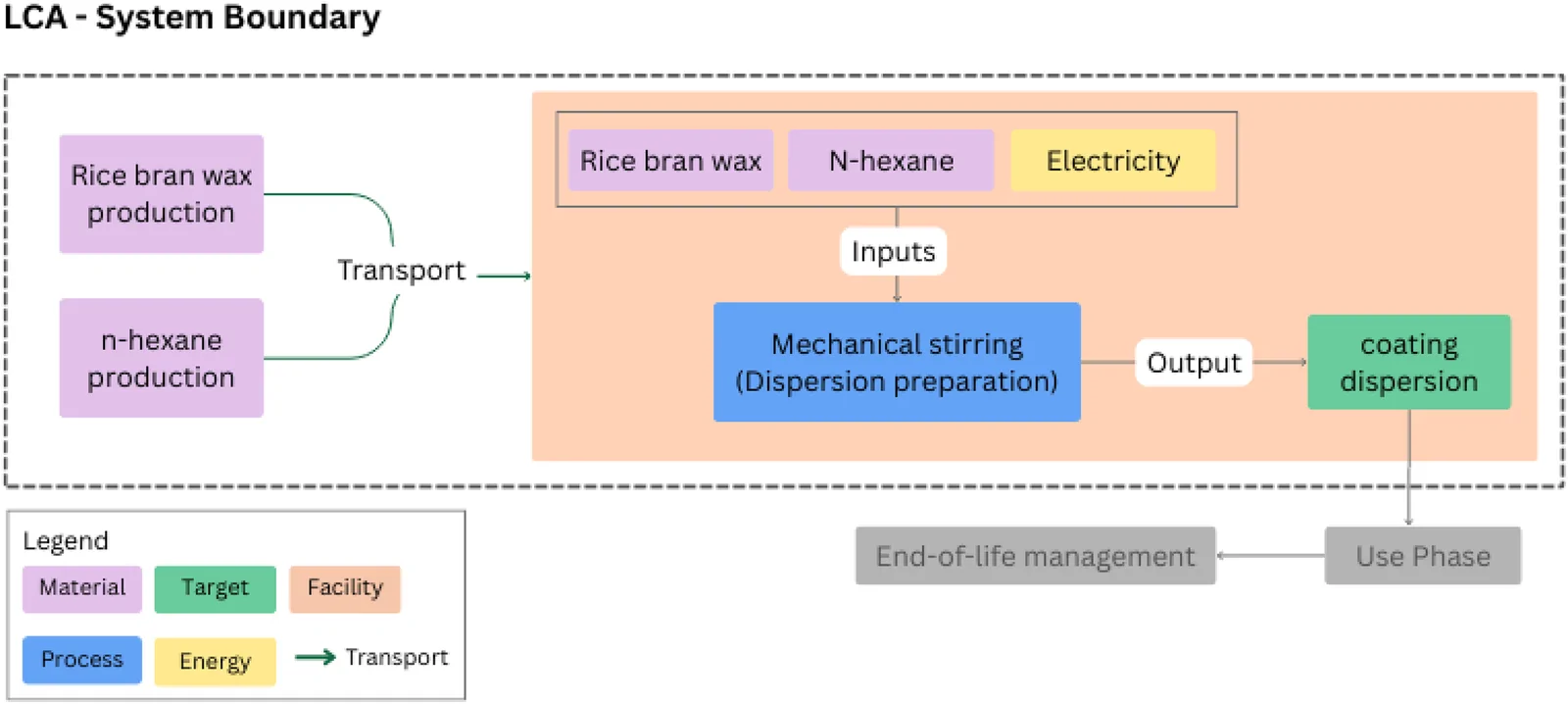
Cradle-to-gate LCA system boundary for the preparation of the rice bran wax–*n*-hexane coating dispersion, including raw material production, transport, and dispersion preparation. The prepared coating dispersion is the foreground output of the assessed system.

## 3. Life cycle inventory

In this section, the life cycle inventory for the preparation of the quantity of rice bran wax–*n*-hexane coating dispersion corresponding to the functional unit is presented. The inventory includes the raw material inputs, their transportation to the facility, energy consumed during dispersion preparation and the foreground output (the coating dispersion).

The LCA was modelled using LCA For Experts (GaBi) 10.9.3.0 (ref. 17) with the Ecoinvent 3.11 (ref. 18) as the primary background database. Foreground inventory data were collected directly from the laboratory where the coating dispersion was prepared, including material quantities, mass measurements, process parameters, and electricity consumption. Material inputs were based on experimentally determined quantities required to achieve effective coating performance. These values reflect the actual amount of material needed under laboratory conditions, inherently including any process-related losses or handling inefficiencies, rather than representing a theoretical material requirement. Electrical energy use during dispersion preparation was measured in real time using a Smart Plug energy monitoring device (Emporia Energy),^19^ enabling equipment-level quantification under actual operating conditions. Auxiliary energy demands associated with general laboratory infrastructure (*e.g.*, fume hood ventilation and building services) were not attributed to individual process steps and are considered part of background building utilities.

Primary data were prioritized where available, while secondary data from peer-reviewed literature were used to represent background processes. Where direct inventory data were unavailable, proxy datasets were applied (indicated where relevant). In particular, *n*-hexane was modelled using a generic hexane production dataset, and rice bran wax was approximated using a rice bran oil production dataset,^20^ with wax-related impacts derived according to reported wax yields.^21^ Rice bran wax is treated as a co-product of the rice bran oil refining process, as it has established commercial value and downstream applications. Therefore, a mass-based allocation approach was applied to partition environmental burdens among co-products in the baseline model. The detailed calculation procedure used to derive the rice bran wax yield is provided in the SI (S3).

Transportation impacts were modelled from the original sourcing locations of raw materials to the laboratory based on estimated transport distances and using standard assumptions regarding transport modes, vehicle types, and emissions consistent with established LCA practice, representing a reasonable approximation of freight transport in the absence of fully region-specific datasets. The sourcing locations reflect the actual supply chain of materials used in the experimental work. The life cycle inventory for the functional unit is summarized in Table 1.

**Table 1:** Life cycle inventory data corresponding to the preparation of the quantity of rice bran wax–*n*-hexane coating dispersion required for coating 1 m^2^ of glass surface. Inventory data include material inputs, energy use, transportation distances, and the foreground output

Stage	Flow type	Sub-stage	Value (functional unit)	Unit
Raw materials	Input	*N*-hexane	3.0	L
	Input	Rice bran wax	0.045	kg
Transportation		*N*-hexane to the lab		
	Transportation	Leg A (US inland)	40.7	km
	Transportation	Leg B (ocean)	5164.5	km
	Transportation	Leg C (UK inland)	40.5	km
		Rice bran wax to the lab		
	Transportation	Leg D (UK inland)	130	km
Dispersion preparation	Utility	Mechanical stirring (electricity)	3.5	kWh
	Output	Coating dispersion	2.1	kg

## 4. Life cycle impact assessment

For the life cycle impact assessment (LCIA) phase, the environmental performance of each material and process involved in the dispersion preparation was quantified using the CML (2001 – August 2016) methodology as implemented in GaBi, which corresponds to the latest available update of the CML method. This method was selected to ensure methodological consistency and comparability with the reference study from which the environmental impact data for rice bran wax were sourced, which applied the CML 2001 (January 2016) version. Both versions follow the same methodological framework and impact category definitions, although the August 2016 update includes minor revisions to certain characterization factors. Consequently, small numerical differences may occur between results generated with the two versions, but these differences are not expected to affect the overall interpretation or comparability of the results.

The midpoint impact categories available and relevant within the selected CML implementation were considered in the assessment to provide a comprehensive evaluation of potential environmental impacts. The environmental impact categories assessed were Acidification Potential (AP), Global Warming Potential (GWP), Freshwater Aquatic Ecotoxicity Potential (FAETP), Marine Aquatic Ecotoxicity Potential (MAETP), Terrestrial Ecotoxicity Potential (TETP), Abiotic Depletion Potential – fossil fuels (ADP fossil), Eutrophication Potential (EP), Human Toxicity Potential (HTP), Abiotic Depletion Potential – elements ultimate reserves (ADP elements), Ozone Layer Depletion Potential (ODP) and Photochemical Oxidation Creation Potential (POCP).

Table 2 presents the characterized results for the selected impact categories, disaggregated by key inputs and processes, including material production (*n*-hexane and rice bran wax), transportation, and electricity consumption for the defined functional unit. To improve clarity and facilitate interpretation, transportation results from all supply routes of the dispersion preparation were aggregated into a single item and grouped accordingly. This aggregation enables a clearer comparison of the relative contribution of each main component to the overall environmental profile. It should be noted that direct comparisons between impact categories should be interpreted cautiously, as they are expressed in different units and rely on distinct characterization factors.

**Table 2:** Characterized LCIA results per functional unit, disaggregated by material inputs, aggregated transport, and electricity consumption.

Impact category	*N*-hexane	Rice bran wax	Transportation	Electricity
Abiotic depletion potential (elements) (kg Sb eq.)	1.91 × 10^−5^	1.67 × 10^−8^	2.11 × 10^−7^	1.54 × 10^−5^
Abiotic depletion potential (fossil) (MJ)	1.22 × 10^2^	9.14 × 10^−1^	1.70 × 10^0^	1.50 × 10^1^
Acidification potential (kg SO_2_ eq.)	8.26 × 10^−3^	1.05 × 10^−3^	2.53 × 10^−3^	2.99 × 10^−3^
Eutrophication potential (kg phosphate eq.)	7.76 × 10^−3^	1.85 × 10^−4^	3.62 × 10^−4^	1.26 × 10^−3^
Freshwater aquatic ecotoxicity potential (kg DCB eq.)	1.15 × 10^0^	1.29 × 10^−4^	2.68 × 10^−2^	1.13 × 10^0^
Global warming potential (kg CO_2_ eq.)	2.41 × 10^0^	9.24 × 10^−2^	1.35 × 10^−1^	1.06 × 10^0^
Human toxicity potential (kg DCB eq.)	2.29 × 10^0^	1.60 × 10^−3^	7.47 × 10^−2^	1.56 × 10^0^
Marine aquatic ecotoxicity potential (kg DCB eq.)	1.78 × 10^3^	1.09 × 10^0^	4.54 × 10^1^	1.13 × 10^3^
Ozone depletion potential (kg R11 eq.)	1.70 × 10^−7^	0.00 × 10^0^	1.93 × 10^−9^	3.74 × 10^−8^
Photochemical ozone creation potential (kg ethene eq.)	7.07 × 10^−3^	7.67 × 10^−5^	1.74 × 10^−4^	2.88 × 10^−4^
Terrestrial ecotoxicity potential (kg DCB eq.)	2.36 × 10^−2^	1.66 × 10^−5^	9.84 × 10^−4^	6.91 × 10^−3^

As shown in Table 2, *n*-hexane contributes the highest characterized impact in all assessed categories, whereas rice bran wax consistently contributes the lowest. Electricity consumption represents the second largest contributor, while transportation contributes comparatively little to the overall environmental profile. A detailed interpretation of these contribution patterns is provided in Section 5.

## 5. Interpretation

Building on the characterized results presented in Table 2, this section analyzes the environmental impact values of key inputs and processes involved in the dispersion preparation process to identify those that contribute most significantly to the overall environmental footprint. In addition, the distribution of impacts across the categories highlights which environmental impact categories are most strongly influenced by the different inputs and processes. All results discussed in this section are expressed relative to the defined functional unit, ensuring consistency in the comparison of inputs and processes. Fig. 3 presents the percentage contribution of each input to each environmental impact category.

**Fig. 3:**
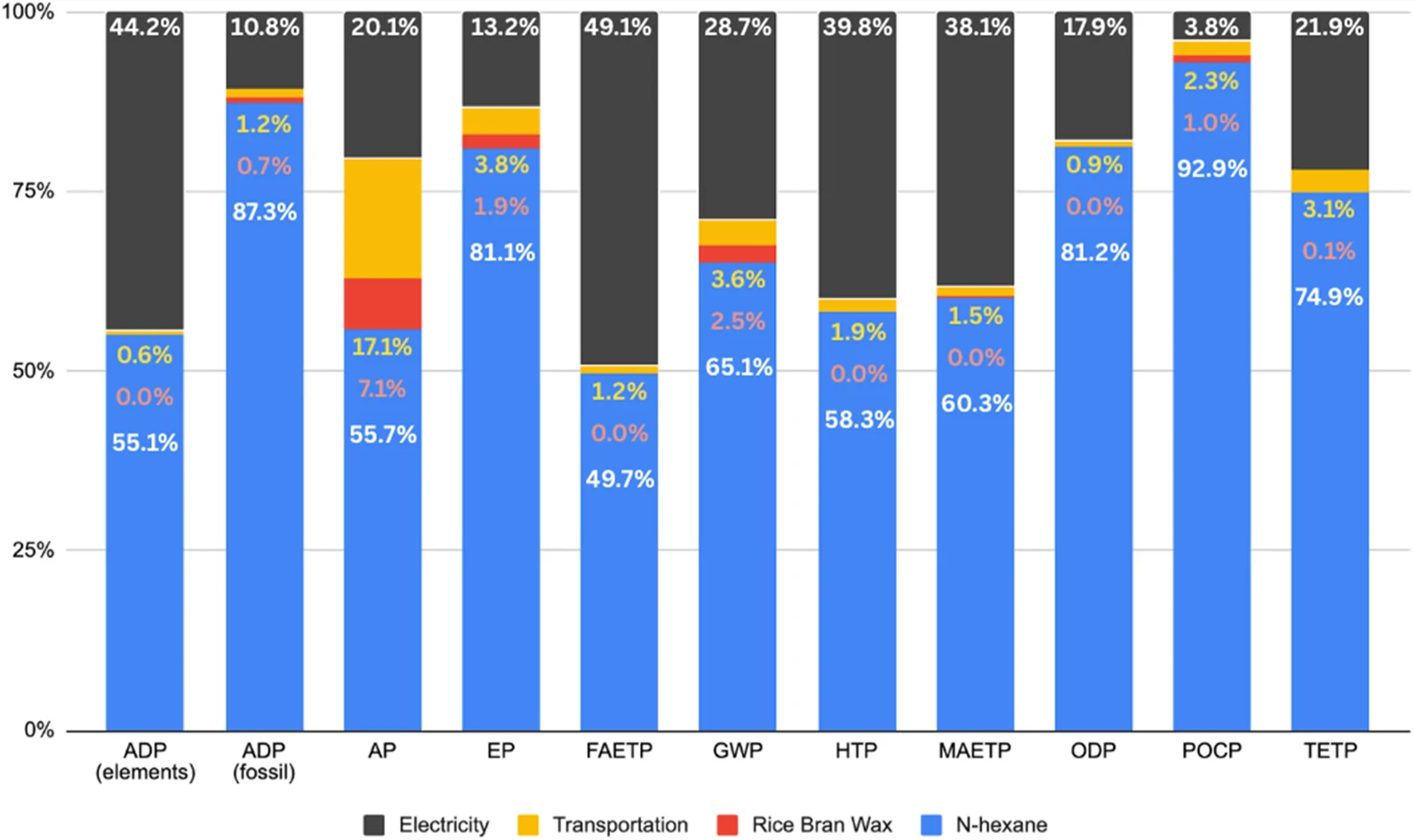
Relative contribution (%) of material inputs, transport, and electricity to CML impact categories per functional unit.

*N*-hexane dominates the environmental profile across all impact categories, with contributions ranging from 49.7% in FAETP to 92.9% in POCP. Its high contribution to POCP reflects the upstream emissions associated with *n*-hexane production represented in the background life cycle inventory. As a VOC, *n*-hexane reacts with nitrogen oxides in the atmosphere in the presence of sunlight, producing ground-level ozone, a key component of photochemical smog.^22^ However, direct solvent evaporation during coating application is outside the defined system boundary of the present study. Similarly, the elevated contribution to ODP (81.2%) indicates the relevance of upstream chemical manufacturing and energy production processes associated with *n*-hexane production, which contribute to emissions linked to stratospheric ozone layer depletion. A substantial contribution was also observed in ADP (fossil) (87.3%), reflecting the dependence of *n*-hexane production on petroleum extraction, refining, and fossil-based feedstocks. High contributions to EP (81.1%) and TETP (74.9%) further suggest that upstream industrial activities associated with solvent production contribute significantly to nutrient-related emissions and toxic releases that affect terrestrial ecosystems. Moderately high contributions from *n*-hexane were additionally observed in GWP (65.1%), MAETP (60.3%), HTP (58.3%), AP (55.7%), ADP (elements) (55.1%), and FAETP (49.7%). Collectively, these findings suggest that the environmental burden of *n*-hexane is primarily associated with upstream petrochemical supply chains rather than its direct use alone, emphasizing the influence of fossil resource extraction, refining operations, and energy-intensive solvent production across multiple environmental impact categories. These findings identify *n*-hexane production and associated upstream petrochemical processes as the primary environmental hotspot within the defined system boundary.

Electricity consumption was identified as the second most significant contributor to the environmental profile of the dispersion preparation process. Unlike *n*-hexane, whose impacts are primarily associated with upstream emissions from solvent production and fossil feedstock dependence, electricity contributions were more pronounced in toxicity- and resource-related categories, particularly FAETP (49.1%), ADP (elements) (44.2%), HTP (39.8%), and MAETP (38.1%). This contribution pattern suggests that electricity-related impacts are primarily driven by the energy-intensive nature of the dispersion preparation step and the upstream burdens embedded within the electricity generation mix represented in the background dataset, which includes fossil-based, nuclear, and renewable energy sources. The comparatively lower contributions observed in POCP (3.8%) and ADP (fossil) (10.8%) further indicate that electricity plays a secondary role in atmospheric VOC-related impacts and fossil feedstock depletion relative to *n*-hexane. Electricity consumption in this study was modeled using the Ecoinvent 3.11 dataset “market for electricity, low voltage”, which represents the UK regional electricity supply mix and includes upstream processes associated with electricity generation and distribution.^23^ The environmental impacts associated with electricity consumption were assumed to scale proportionally with the experimentally measured energy demand, following the definition of the functional unit and the assumption of linear scaling from laboratory conditions.

Transportation plays a relatively minor role in the overall environmental impacts of the system. The highest contribution is observed in AP (17.1%), likely associated with acidifying emissions generated during fuel combustion in transportation processes. Contributions to all other impact categories remained below 4%, indicating that transport-related emissions play a comparatively limited role in the overall environmental performance of the system.

Rice bran wax contributes marginally across all impact categories, with most values remaining below 3%. This indicates a limited influence of its upstream production processes on the overall environmental profile of the system.

Under these assumptions, *n*-hexane production and its associated upstream petrochemical supply chains emerged as the dominant contributors across all impact categories, reflecting the substantial environmental burdens associated with fossil resource extraction, refining operations, and solvent manufacture. Electricity demand represented the main secondary hotspot, while transportation and rice bran wax contributed only marginally to the overall environmental profile. These results highlight the strong sensitivity of the system to solvent selection. Accordingly, a sensitivity analysis was conducted to evaluate the influence of key parameters, including solvent selection, electricity consumption, and allocation approach, on the overall environmental results.

## 6. Parameter sensitivity analysis

### 6.1. Solvent alternative

A sensitivity analysis was performed to evaluate the influence of the solvent selection on the environmental performance of the dispersion preparation process, as its production and associated upstream processes were identified as the main environmental hotspot.

In this analysis, *n*-hexane was replaced with petroleum ether, a proxy derived from a naphtha-based hydrocarbon fraction. Petroleum ether was selected as an alternative solvent because it was identified in the coating fabrication study as a potential substitute for *n*-hexane.^13^ Although its use resulted in slightly reduced coating performance, it was still considered suitable for the fabrication process, making it a relevant alternative for evaluating potential environmental improvements. It should be noted that the use of proxy datasets for both solvents introduces some uncertainty. However, this comparison provides a reasonable approximation of the relative environmental implications associated with solvent substitution. The solvent quantity was kept constant, as the referenced study indicates the same quantity is required for both solvents.^13^ All other modelling assumptions, including electricity consumption, the defined functional unit, and system boundaries, were kept constant.

Fig. 4 presents the relative contribution of the different inputs across the environmental impact categories under the petroleum ether substitution scenario. Compared with the baseline scenario, the relative contribution of the solvent decreases across all impact categories, leading to a more balanced distribution between solvent- and electricity-related impacts. Fig. 5 illustrates how changes in the solvent selection affect its relative contribution to each environmental impact category. All values are expressed relative to the baseline solvent scenario, which is normalized to 1, so deviations indicate proportional increases or reductions compared to that reference. In particular, for TETP, POCP, MAETP, HTP, FAETP, EP, and ADP (elements), the relative contribution of the solvent decreases to below 50% when petroleum ether is used. Among these categories, MAETP, HTP, FAETP, EP, and ADP (elements) show even more pronounced reductions, with normalized solvent contributions falling below 25%. This highlights the strong dependence of these impact categories on solvent selection. Intermediate reductions are found in AP and GWP where the solvent still accounts for 52% and 58%, respectively, of the original impact within those categories. For ADP (fossil) and ODP, the relative contribution of petroleum ether remains comparatively high, with values close to 80%, suggesting that these categories are less sensitive to solvent substitution in terms of contribution structure, despite moderate reductions in absolute impacts. Overall, these results indicate that replacing *n*-hexane with petroleum ether reduces the environmental burden associated with solvent use. These results confirm the dominant role of the solvent selection in driving the environmental impacts of the dispersion preparation process and demonstrate how hotspot identification through life cycle assessment can support the evaluation of alternative sourcing options.

**Fig. 4:**
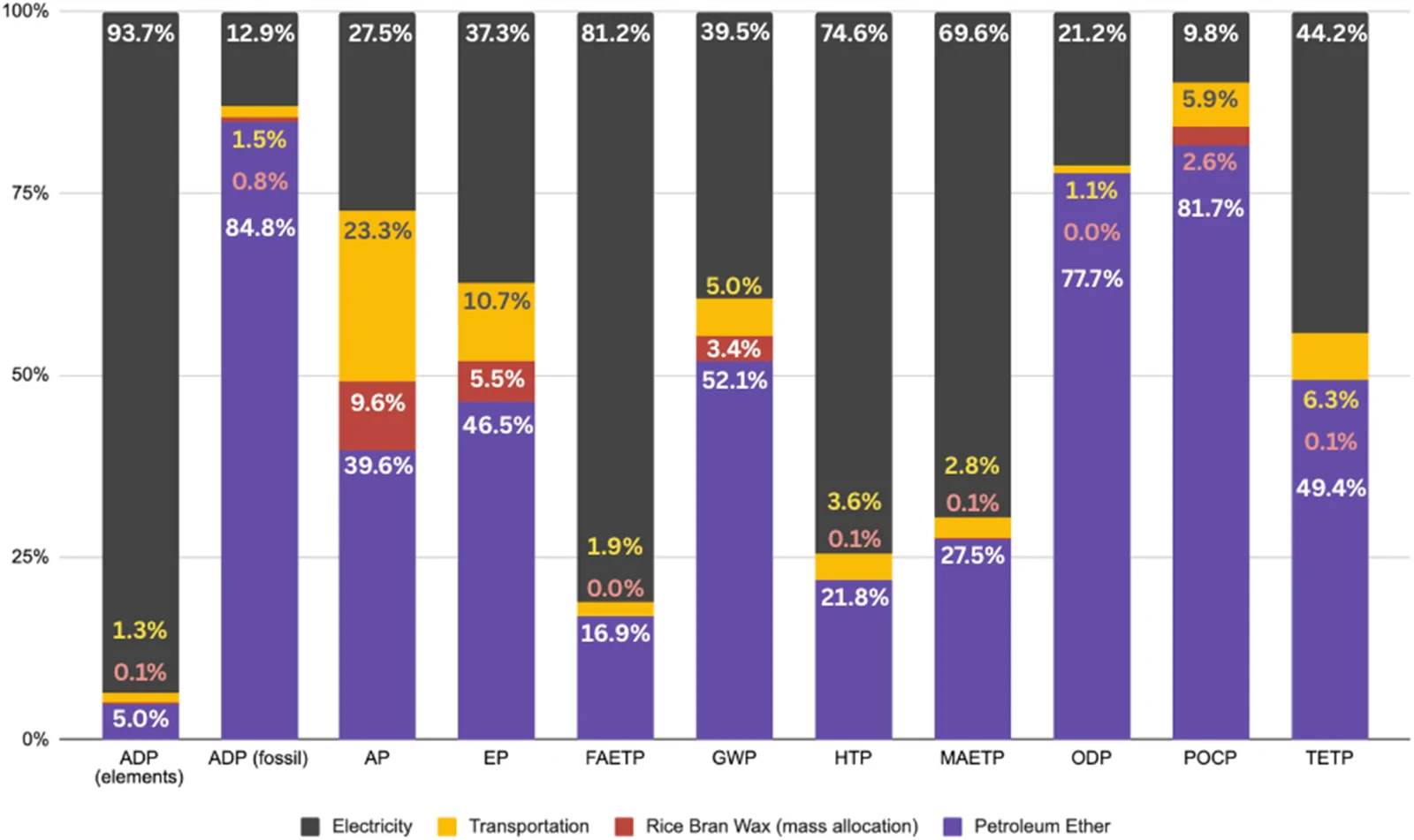
Relative contribution (%) under the petroleum ether substitution scenario of material inputs, transport, and electricity to CML impact categories per functional unit.

**Fig. 5:**
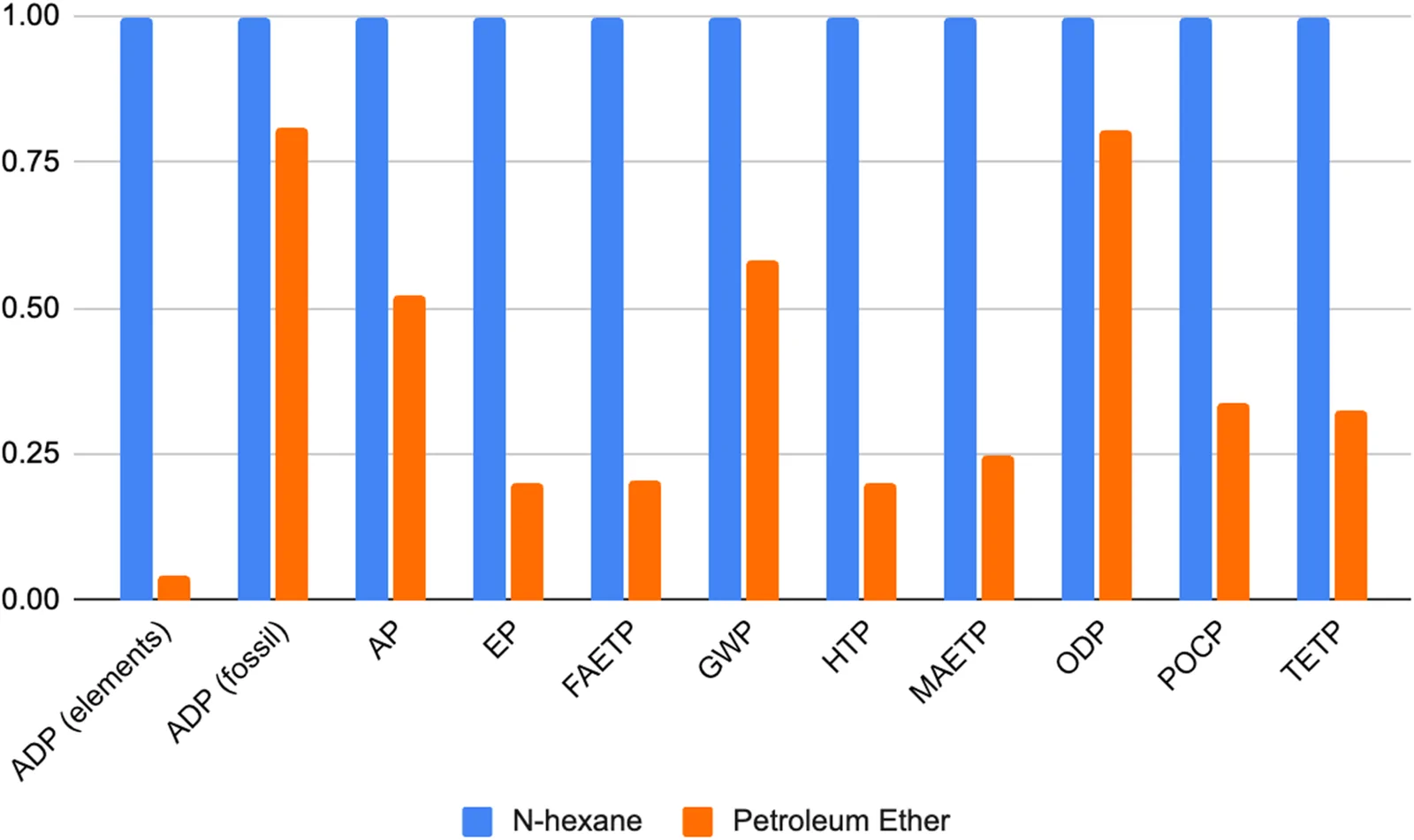
Relative contribution of *n*-hexane and petroleum ether to each CML impact category expressed as a ratio normalized to the baseline *n*-hexane solvent case.

Table 3 summarizes the characterized LCIA results for the dispersion preparation process under the *n*-hexane and the petroleum ether scenario. Overall, replacing *n*-hexane with petroleum ether leads to consistent reductions across all impact categories, with the magnitude of the reductions ranging from 16% (ODP) to 64% (EP). The largest reductions were observed for EP (64%), followed by POCP (61%), ADP (elements) (53%), and TETP (51%). Intermediate reductions were observed for HTP (47%), MAETP (46%) and FAETP (40%), whereas GWP (26%), AP (22%), ADP (fossil) (17%) and ODP (16%) exhibit comparatively smaller reductions. Despite the smaller reductions observed for some impact categories, petroleum ether consistently outperformed *n*-hexane across all categories considered. Although petroleum ether was reported to result in slightly lower coating performance compared with *n*-hexane,^13^ the environmental assessment demonstrates substantial reductions across all impact categories. This highlights the importance of integrating life cycle assessment into material selection and process design decisions, as modest reductions in technical performance may be offset by significant environmental improvements.

**Table 3:** Characterized LCIA results per functional unit comparing *n*-hexane and petroleum ether scenarios, with percentage reduction

Impact category	LCIA total (baseline)	LCIA total (petroleum ether)	Reduction from baseline value
Abiotic depletion potential (elements)	3.51 × 10^−5^	1.64 × 10^−5^	53%
Abiotic depletion potential (fossil)	1.41 × 10^2^	1.16 × 10^2^	17%
Acidification potential	1.40 × 10^−2^	1.09 × 10^−2^	22%
Eutrophication potential	9.43 × 10^−3^	3.38 × 10^−3^	64%
Freshwater aquatic ecotoxicity potential	2.32 × 10^0^	1.39 × 10^0^	40%
Global warming potential	3.65 × 10^0^	2.69 × 10^0^	26%
Human toxicity potential	3.97 × 10^0^	2.10 × 10^0^	47%
Marine aquatic ecotoxicity potential	2.98 × 10^3^	1.62 × 10^3^	46%
Ozone depletion potential	2.09 × 10^−7^	1.76 × 10^−7^	16%
Photochemical ozone creation potential	7.55 × 10^−3^	2.94 × 10^−3^	61%
Terrestrial ecotoxicity potential	3.19 × 10^−2^	1.56 × 10^−2^	51%

### 6.2. Electricity alternative

Given that the electricity supply mix represents the second most relevant contributor across several impact categories, an additional sensitivity analysis considering an alternative electricity supply mix was performed.

In this analysis, the baseline electricity dataset was replaced with data representing electricity supplied exclusively from renewable energy technologies. As no equivalent renewable energy dataset for the United Kingdom was available in our selected database (Ecoinvent), the Swiss dataset was used as the closest proxy to illustrate the potential effects of sourcing electricity from predominantly renewable energy systems. It should be noted that differences between the Swiss and UK electricity systems may introduce some uncertainty. However, the scenario is intended to represent a general shift towards low-carbon electricity rather than a geographically exact substitution. It should also be noted that changes in relative contribution do not necessarily correspond to proportional changes in total environmental impacts, which are assessed separately through the characterized LCIA results. All other modelling assumptions were kept constant. The sensitivity analysis was performed under the petroleum ether substitution scenario to ensure consistency with the previous sensitivity analysis.

Fig. 6 illustrates how changes in the electricity supply mix affect its relative contribution to each environmental impact category. Across all categories, the substitution leads to a systematic reduction in the relative contribution of electricity. In particular, for ADP (fossil), AP, EP, GWP, ODP, and POCP, the relative contribution of electricity decreases to below 50% when renewable electricity is used. Among these categories, ADP (fossil), GWP, ODP, and POCP show even more pronounced reductions, with electricity contributions falling below 25%. This highlights the strong dependence of these impact categories on the carbon intensity of the electricity supply.

**Fig. 6:**
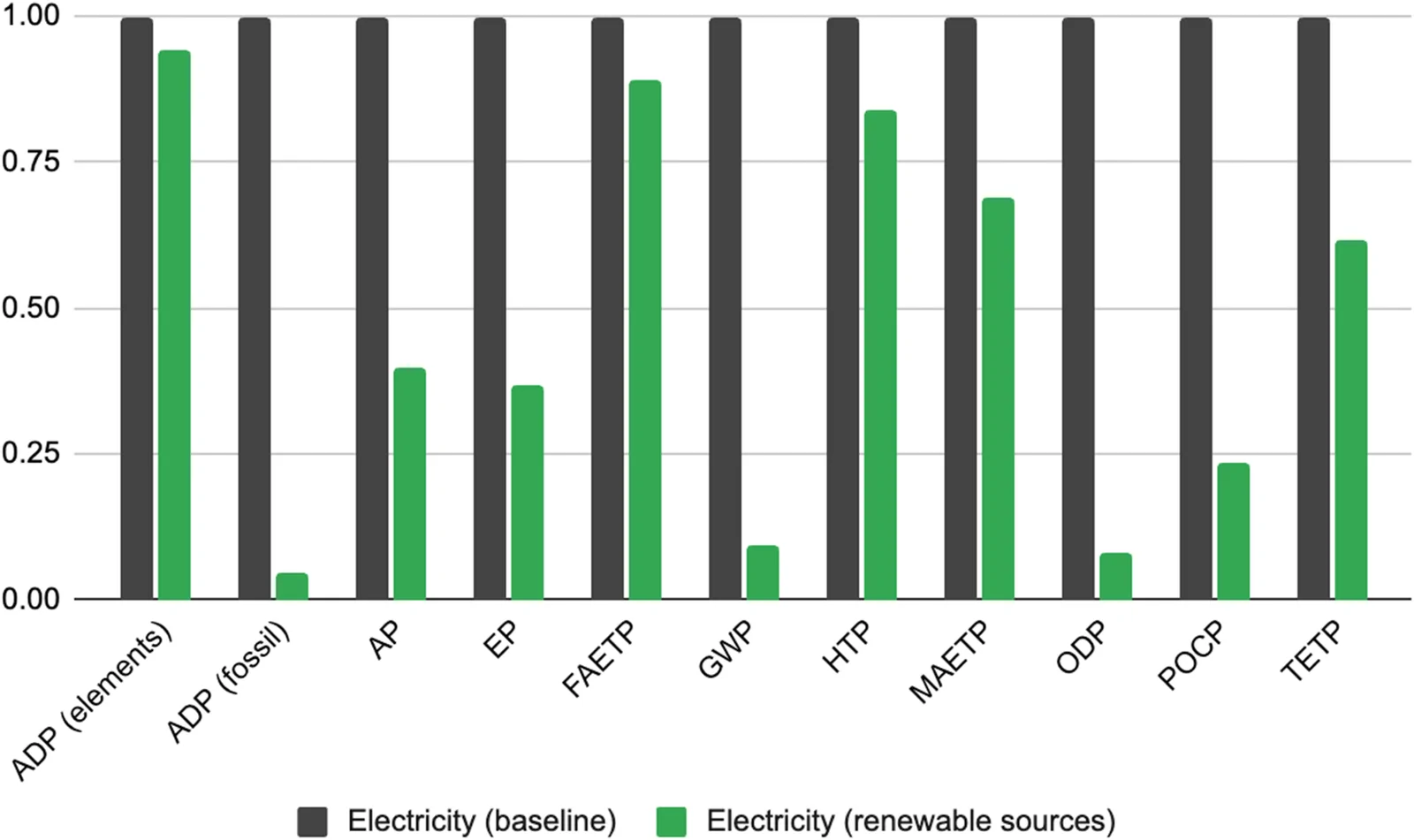
Relative contribution of electricity in baseline and renewable scenarios to each CML impact category, expressed as a ratio normalized to the baseline electricity case.

Table 4 summarizes the characterized LCIA results for the dispersion preparation process under the baseline electricity mix and the renewable electricity scenario, under the petroleum ether substitution scenario. Overall, replacing the electricity supply mix leads to consistent reductions across all impact categories, in particular GWP exhibits a variation of 36%.

**Table 4:** Characterized LCIA results per functional unit comparing baseline and renewable electricity scenarios (Swiss mix) under the petroleum ether scenario, with percentage reduction

Impact category	LCIA total (energy baseline)	LCIA total (renewable electricity)	Reduction from baseline value
Abiotic depletion potential (elements)	1.64 × 10^−5^	1.55 × 10^−5^	5%
Abiotic depletion potential (fossil)	1.16 × 10^2^	1.02 × 10^2^	12%
Acidification potential	1.09 × 10^−2^	9.08 × 10^−3^	16%
Eutrophication potential	3.38 × 10^−3^	2.58 × 10^−3^	24%
Freshwater aquatic ecotoxicity potential	1.39 × 10^0^	1.27 × 10^0^	9%
Global warming potential	2.69 × 10^0^	1.73 × 10^0^	36%
Human toxicity potential	2.10 × 10^0^	1.84 × 10^0^	12%
Marine aquatic ecotoxicity potential	1.62 × 10^3^	1.27 × 10^3^	22%
Ozone depletion potential	1.76 × 10^−7^	1.41 × 10^−7^	20%
Photochemical ozone creation potential	2.94 × 10^−3^	2.72 × 10^−3^	7%
Terrestrial ecotoxicity potential	1.56 × 10^−2^	1.30 × 10^−2^	17%

### 6.3. Wax allocation alternative

An additional sensitivity analysis was conducted to evaluate the influence of the rice bran wax allocation approach on the environmental results, given the multi-functionality of the rice bran oil refining process and the need to partition environmental burdens among co-products. An alternative allocation method based on the economic value of rice bran oil co-products was applied. Market price and revenue data for rice bran oil processing products were obtained from Kabir *et al.*,^24^ who reported sales prices and annual revenues for oil, de-oiled rice bran, fatty acids, wax, gum, and splint earth in a Bangladeshi rice bran oil mill. Although these data are region- and time-specific (Bangladesh, 2011), they were considered a reasonable proxy in the absence of more recent or regionally representative datasets. In that study, rice bran wax accounts for 1% of the total product value of the refinery outputs. Therefore, 1% of the environmental burdens of the rice bran oil processing system were assigned to rice bran wax under economic allocation. Detailed calculations are included in the SI (S3). Given that the contribution of rice bran wax was already below 1% across all impact categories under mass allocation, its reduction to approximately one-third under economic allocation further diminishes its influence on the overall environmental profile, rendering its contribution practically negligible within the context of the overall environmental profile.

Table 5 presents the characterized LCIA results under the combined sensitivity scenario, integrating solvent substitution, changes in electricity supply, and rice bran wax allocation method, and comparing them with the baseline results. Overall, the combined scenario leads to substantial reductions across all impact categories, with the most significant decreases observed in EP (74%), POCP (65%), TETP (59%), MAETP (57%), GWP and ADP (elements) (55%) and HTP (53%). These results confirm that the combined implementation of the analyzed alternatives reduces the contribution to all the assessed environmental impact categories, primarily driven by solvent substitution and electricity decarbonization, while changes in allocation have a comparatively minor influence on the overall environmental performance.

**Table 5:** Characterized LCIA results per functional unit comparing baseline and combined sensitivity scenario (petroleum ether substitution, renewable electricity, and wax economic allocation), with percentage reduction

Impact category	LCIA total (baseline)	LCIA total (renewable electricity, petroleum ether, and rice bran wax economic allocation)	Reduction from baseline value
Abiotic depletion potential (elements)	3.47 × 10^−5^	1.55 × 10^−5^	55%
Abiotic depletion potential (fossil)	1.39 × 10^2^	1.01 × 10^2^	27%
Acidification potential	1.48 × 10^−2^	8.39 × 10^−3^	43%
Eutrophication potential	9.56 × 10^−3^	2.46 × 10^−3^	74%
Freshwater aquatic ecotoxicity potential	2.30 × 10^0^	1.27 × 10^0^	45%
Global warming potential	3.71 × 10^0^	1.67 × 10^0^	55%
Human toxicity potential	3.93 × 10^0^	1.84 × 10^0^	53%
Marine aquatic ecotoxicity potential	2.95 × 10^3^	1.27 × 10^3^	57%
Ozone depletion potential	2.09 × 10^−7^	1.41 × 10^−7^	32%
Photochemical ozone creation potential	7.61 × 10^−3^	2.67 × 10^−3^	65%
Terrestrial ecotoxicity potential	3.15 × 10^−2^	1.30 × 10^−2^	59%

Fig. 7 provides a final overview of the distribution of environmental impacts under the combined sensitivity scenario, integrating changes in solvent selection, electricity supply, and rice bran wax allocation method, while allowing a comparison with the baseline scenario initially analysed (Fig. 3).

**Fig. 7:**
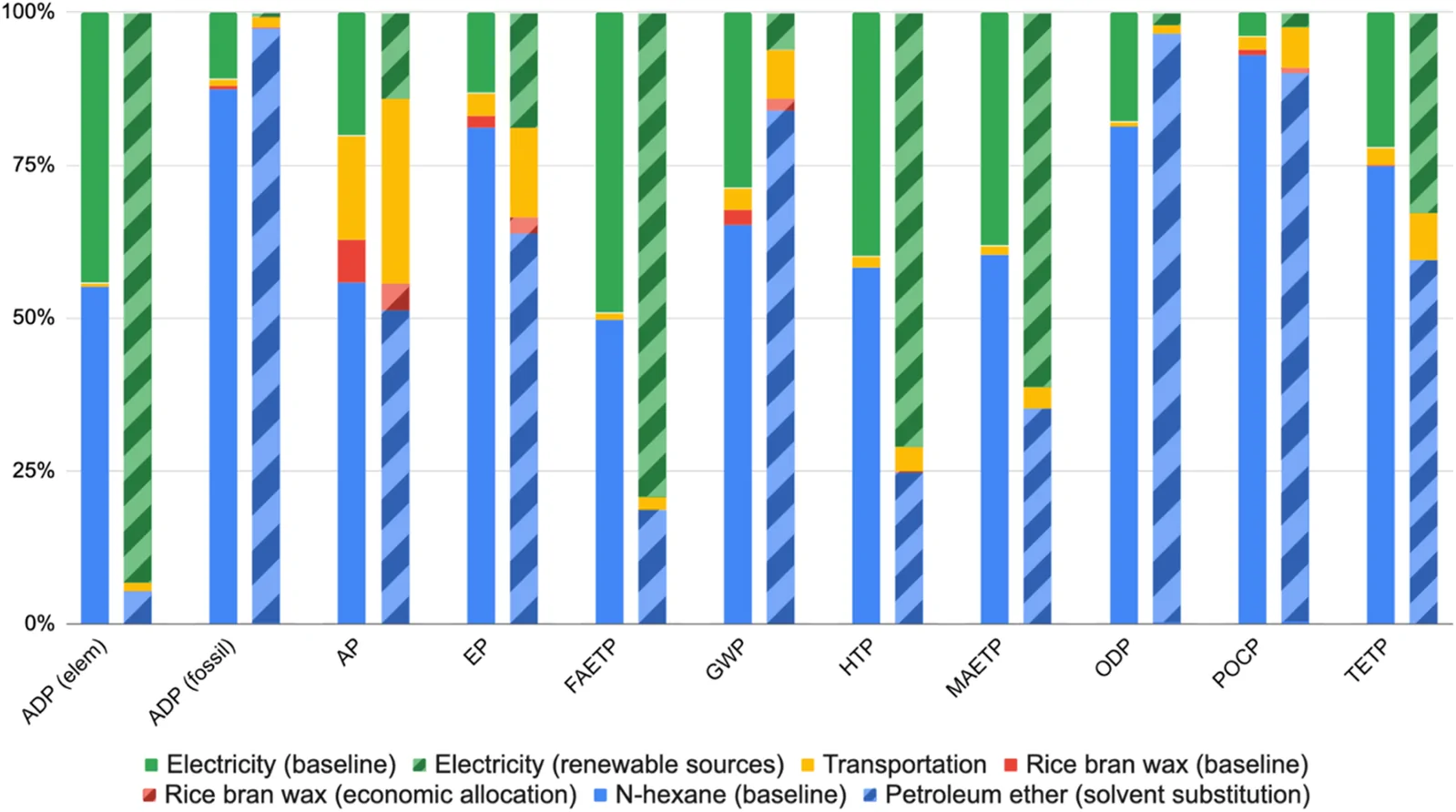
Relative contribution (%) of inputs to CML impact categories for the baseline (solid bars) and combined sensitivity scenario (striped bars), including petroleum ether substitution, renewable electricity, and economic allocation of rice bran wax.

This illustrates how the combined implementation of process improvements not only reduces the overall environmental impacts of the system (as can be observed in Table 5) but also redistributes environmental burdens among inputs, leading to changes in the identity of the dominant contributors in several impact categories. This is particularly evident in ADP (elements), FAETP, HTP and MAETP, where the reduction in solvent-related impacts increases the relative importance of electricity-related processes within the overall contribution profile.

## 7. Limitations, challenges and perspectives

Several limitations should be considered when interpreting the results of this LCA. First, some foreground inputs were represented using proxy datasets due to the lack of specific life cycle inventory data. In particular, *n*-hexane was modelled using the generic hexane production dataset from the Ecoinvent database, which represents a mixture of hexane isomers (C_6_H_14_) classified under the CAS number 110-54-3. Although this dataset includes *n*-hexane, the exact composition of individual isomers is not specified, which may introduce uncertainty in the representation of the solvent production process.^25^

Similarly, rice bran wax production was approximated using rice bran oil production data, with wax-related impacts estimated based on reported wax yields. Literature sources indicate that rice bran wax typically represents approximately 2–4% of rice bran oil production,^21^ and a value of 3% was applied in this study. Variations in wax yield across production systems may therefore influence the estimated environmental impacts associated with this material. Moreover, the allocation of environmental burdens among co-products introduces methodological uncertainty, as different allocation approaches (*e.g.*, mass-based or economic) can lead to different results. The detailed calculation procedure used to derive the impacts associated with rice bran wax is provided in the SI (S3).

Additional assumptions were required for transportation modelling, as detailed logistical data regarding transport routes and shipment conditions were not available. Transport distances were estimated using online mapping tools^26^ and maritime distance calculators^27^ based on the known origin and destination locations of the raw materials. Land transportation in the United States was modelled using heavy-duty diesel trucks (16–32 t) based on the closest available dataset (Brazil), while transatlantic transport between the United States and the United Kingdom was represented using container ship transport powered by heavy fuel oil. Land transportation within the United Kingdom was modelled using heavy-duty EURO 6 trucks based on the closest available regional dataset (RER). While these assumptions are consistent with established LCA practice, actual transportation conditions may differ.

Another limitation relates to the estimation of electricity consumption for the mechanical stirring step. This step was estimated based on laboratory-scale measurements and normalized to the functional unit. The values were linearly scaled up with process electricity consumption, following common practice in early-stage LCA studies of coating systems.^8–10^ However, this approach assumes proportional energy demand across scales and does not capture potential changes in industrial-scale conditions, where mixing efficiency, equipment design, and process integration may significantly influence energy consumption. Therefore, the reported values should be interpreted as representative of laboratory-scale conditions rather than full-scale manufacturing, and the dominance of electricity should be understood within the context of these scaling assumptions.

Additional limitations relate to the datasets used in the sensitivity analyses. The solvent sensitivity analysis used a petroleum ether proxy derived from a naphtha-based hydrocarbon fraction, as no specific life cycle inventory dataset for petroleum ether was available in the Ecoinvent database used in this study. Furthermore, the renewable electricity scenario was modelled using a Swiss electricity mix, as no equivalent renewable energy dataset was available for the United Kingdom within the selected database (Ecoinvent), where the laboratory experiments were conducted. More broadly, the use of datasets from different geographical regions and time periods (*e.g.*, Switzerland, Bangladesh, and global averages) may introduce variability due to differences in energy systems, technologies, and market conditions. These assumptions introduce some uncertainty in the estimated impacts of the alternative scenarios.

In addition, the study adopts a cradle-to-gate system boundary and therefore does not account for the use and end-of-life phases of the coating, which may influence the overall environmental performance depending on application conditions and disposal pathways. Furthermore, although glass was selected as a reproducible model substrate to enable consistent assessment of coating fabrication, the present analysis does not account for substrate-specific interactions that may influence the environmental performance of coated paper, fibre-based materials, or other food-packaging substrates. Evaluating complete coated packaging systems across their full life cycle therefore remains an important area for future research. Accordingly, direct solvent evaporation during spray application, associated VOC emissions, solvent recovery, and solvent waste management were not evaluated because these processes occur after dispersion preparation and therefore fall outside the defined cradle-to-gate system boundary. These downstream processes should be considered in future cradle-to-grave assessments of the complete coating system.

Despite these limitations, the modelling assumptions applied are consistent with established life cycle assessment practices and are considered sufficient to support the identification of the main environmental hotspots associated with the dispersion preparation process.

As previously mentioned, assessing the environmental impact of an emerging food-packaging coating represents only one component of a broader sustainability assessment. As this coating is currently in the early development stages, its biodegradability, compostability, recyclability and food-contact suitability have not yet been evaluated. However, a preliminary discussion of these aspects is presented below.

Because the coating formulation uses a VOC as a carrier, appropriate application conditions are expected to promote solvent evaporation during spray deposition, resulting in minimal residual VOCs in the finished coating. Consequently, the deposited coating is expected to consist primarily of rice bran wax. Therefore, the following discussion focuses on the sustainability aspects of rice bran wax.

First, rice bran wax is obtained as a by-product of rice milling and rice bran oil refining.^21^ Its utilization is, therefore, consistent with circular economy principles by valorizing an agricultural co-product rather than requiring the production of a dedicated raw material. Second, as a naturally derived plant wax, rice bran wax is expected to be biodegradable under appropriate environmental conditions. However, compostability cannot be inferred from biodegradability alone and should be demonstrated through testing against recognized standards, such as ISO 17088:2021.^28^

From the perspective of packaging recyclability, even if the coating itself is ultimately shown to be biodegradable, an important consideration is the ease with which it can be removed from the packaging substrate. Future work should focus on developing coatings that provide sufficient adhesion and durability during use while remaining readily removable at the end of their service life, thereby facilitating the independent recovery and recycling of each component. Such an approach would help minimize disruption to existing recycling processes for conventional packaging materials.

Finally, regarding food-contact suitability, refined rice bran wax is permitted by the U.S. FDA for specific direct food applications, including coatings for fruits, vegetables, and confectionery.^29^ Moreover, the European Food Safety Authority concluded that oxidized rice bran wax and its calcium salt do not pose a safety concern for consumers under the specific food-contact conditions evaluated.^30^ Consequently, although food-contact compliance should be demonstrated for the specific coating formulation and intended application, these regulatory assessments provide encouraging evidence supporting the potential use of rice bran wax-based coatings in food-contact applications.

Looking beyond the present study, several challenges remain for the broader implementation of bio-based superhydrophobic coating technologies. Future work should focus on developing application-specific life cycle inventory data, validating the environmental hotspots identified in this study under laboratory- and industrial-scale operating conditions, and expanding the system boundary to include the use and end-of-life phases. In addition, integrating environmental assessment with the broader sustainability considerations discussed above, together with coating performance, durability, and other application-specific requirements, would enable a more comprehensive assessment of the overall sustainability of emerging barrier coating technologies. Such an integrated assessment is necessary because favorable manufacturing-related environmental impacts alone do not guarantee the overall sustainability of food-packaging systems. Future comparative life cycle assessments involving alternative coating formulations and conventional barrier coating technologies would further support the evaluation of emerging bio-based coatings within the broader context of sustainable food-packaging systems. Such efforts would strengthen the role of life cycle assessment as a decision-support tool during material development while facilitating the transition of promising laboratory-scale technologies toward practical implementation.

## 8. Conclusions

This paper presented an environmental analysis of the production of a bio-based superhydrophobic coating developed in previous work.^13^ Based on experimental data, a life cycle inventory was constructed by scaling laboratory-scale inputs to the defined functional unit, enabling the assessment of environmental impacts associated with the dispersion preparation process.

The results indicate that the environmental profile of the dispersion preparation process is primarily driven by solvent selection, which is the dominant contributor across all impact categories. The electricity consumption represents the second most relevant contributor across several impact categories, with notable influence in FAETP, while transportation contributes only marginally across all categories. Rice bran wax plays a negligible role across all impact categories. These findings confirm that solvent production and its associated upstream petrochemical processes represent the primary environmental hotspot of the dispersion preparation process, while electricity acts as a consistent secondary contributor across several impact categories. These conclusions apply specifically to the dispersion preparation stage assessed in this study. Downstream processes, including spray application, solvent evaporation during drying, VOC emissions, solvent recovery, and end-of-life management, were outside the defined system boundary and were therefore not included in the environmental results presented.

The sensitivity analysis further demonstrated that different parameters influence distinct impact categories. Replacing the solvent selection with petroleum ether resulted in substantial reductions in EP, POCP, ADP (elements), and TETP, highlighting the strong influence of solvent production and upstream petrochemical processes on emissions- and toxicity-related impacts. In contrast, changes in the electricity supply mix primarily affected climate- and energy-related categories, particularly GWP. By comparison, the allocation approach applied to rice bran wax had a negligible effect on the overall results, due to its already minimal contribution across all impact categories.

Overall, the combined sensitivity scenario resulted in variations greater than approximately 50% in EP, POCP, TETP, MAETP, ADP (elements), GWP and HTP, while all impact categories showed some degree of improvement, including the smallest variation of approximately 27% observed for ADP (fossil). These results demonstrate that process-related parameters, particularly solvent selection and electricity supply, are key determinants of the coating's environmental profile, as modifications to these inputs result in substantial changes across multiple impact categories.

Beyond the specific case study, these findings illustrate how early-stage life cycle assessment can support the design and development of more sustainable coating systems by enabling the systematic evaluation of material and process alternatives. The environmental baseline established in this study represents one component of the broader sustainability assessment required for emerging food-packaging coatings and should therefore be considered alongside functional performance, food-contact suitability, recyclability, biodegradability, compostability, and other end-of-life considerations. By identifying key environmental drivers and quantifying the effects of targeted modifications, such analyses provide actionable insights that can be integrated into formulation and process design decisions. Although the quantitative results reported in this study are representative of laboratory-scale conditions, the identification of solvent production and electricity supply as the dominant environmental hotspots provides a basis for prioritizing future process optimization during scale-up. In particular, the solvent sensitivity analysis demonstrates how a modest reduction in coating performance may be accompanied by substantial environmental benefits, highlighting the importance of integrating environmental assessment alongside functional performance during the development of emerging coating technologies. Overall, the results indicate that optimizing solvent selection, transitioning to low-impact electricity sources, and appropriately defining allocation methods represent key opportunities to reduce environmental impacts and improve the overall sustainability of the coating dispersion preparation process.

## Conflicts of interest

There are no conflicts to declare.

## Abbreviations

ADP: Abiotic depletion potential
AP: Acidification potential
CAS: Chemical abstracts service
CML: Centrum voor Milieukunde Leiden
EP: Eutrophication potential
FAETP: Freshwater aquatic ecotoxicity potential
FU: Functional unit
GWP: Global warming potential
HTP: Human toxicity potential
ISO: International organization for standardization
LCA: Life cycle assessment
LCIA: Life cycle impact assessment
MAETP: Marine aquatic ecotoxicity potential
ODP: Ozone depletion potential
POCP: Photochemical ozone creation potential
TETP: Terrestrial ecotoxicity potential
VOC: Volatile organic compound

## Supplementary Material

SU-004-D6SU00434B-s001

## Data Availability

The data supporting this article have been included as part of the supplementary information (SI). Supplementary information is available. See DOI: https://doi.org/10.1039/d6su00434b.
