# Supplementary material for: Rice bran wax-hexane superhydrophobic coating production: an environmental assessment
Source: RSC Sustain. 2026 Aug 5;4(10):3678–91. doi: 10.1039/d6su00434b (PMC13644681; doi:10.1039/d6su00434b)
Supplement: Supplementary file 1 [file SU-004-D6SU00434B-s001.pdf]

Supplementary Information (SI) for RSC Sustainability.
This journal is © The Royal Society of Chemistry 2026

Supplementary Information

Section 1 – Experimental parameters used for the coating fabrication process

The coating fabrication procedure is described in Section 2 of the main manuscript and follows the methodology reported by Resendiz-Diaz et al. (13). The parameters relevant for the life cycle inventory are summarized in **Table S1**. These parameters correspond to the coating of a single substrate with a surface area of 0.002 m², which represents the experimental reference flow used to construct the life cycle inventory. The coating solution used in the experiments consisted of rice bran wax dissolved in n-hexane, with a total solution volume of 6 mL per substrate, applied in three successive layers.

Parameter	Value	Unit	Source
Coated surface area	0.002	m^2	Literature (13)
N-hexane in solution	10	mL	Experiment
Rice bran wax in solution	0.15	g	Experiment
Total solution used per sample	6	mL	Experiment
Solution applied per layer	≈ 2	mL	Literature (13)
N-hexane density (20 °C)	0.66	kg L^{-1}	Literature (31)

Table S1. Experimental parameters used to construct the life cycle inventory for the coating fabrication process.

Section 2 – Life cycle inventory for coating preparation

Section 2.1 – Measured electricity consumption

The electricity demand associated with the laboratory-scale preparation of the coating (reference surface area of 0.002 m²) was measured directly during the mechanical stirring process using a smart plug power meter. The device recorded instantaneous power consumption at one-minute intervals throughout the experiment. Total electricity consumption was calculated by summing the recorded power values and multiplying by the sampling interval (1/60 h), yielding the cumulative energy consumption for the process according to:

$$E = \sum_{i=1}^{32} P_i \Delta t$$

where P_i is the instantaneous power recorded at each one-minute interval and $\Delta t = 1/60$ h.

Process	Value
Process	Mechanical stirring
Sampling interval	1 min
Measurement duration	32 min (0.53 h)
Sum of instantaneous power readings (ΣPi)	0.420 kW
Calculated energy consumption	0.007 kWh
Measurement method	Smart Plug Meter

Table S2. Electricity consumption of the mechanical stirring process during laboratory-scale coating preparation.

Section 2.2 – Transportation inventory

These values represent the transportation distances for the raw materials used in the dispersion preparation of the superhydrophobic coating. These distances were estimated based on supplier locations and approximated logistics routes.

Material	Route	From	To	Distance (km)	Mode
N-hexane	Leg A (US inland)	Honeywell International Inc., US	Port Newark–Elizabeth (Port of NY/NJ)	40.7	Truck
N-hexane	Leg B (ocean freight)	Port Newark–Elizabeth (Port of NY/NJ)	DP World London Gateway	5164.5	Ship
N-hexane	Leg C (UK inland)	DP World London Gateway	QMUL, London	40.5	Truck
Rice bran wax	Direct transport	Nutley's Kitchen Gardens UK Ltd	QMUL, London	130	Truck

Table S3. Transportation inventory for raw materials used in the coating dispersion preparation process.

Section 3 – Rice Bran Wax Modelling and Allocation Approaches

Section 3.1 – Mass-based approach (wax yield)

Rice bran wax is obtained as a by-product during the refining of rice bran oil. In this study, the environmental impacts associated with rice bran wax production were approximated using life cycle inventory data for rice bran oil production, combined with literature-reported wax yields. Literature sources indicate that rice bran wax typically represents 2–4% of rice bran oil production (21). Therefore, a midpoint value of 3% was adopted in this study.

Based on this assumption, the production of 1000 kg of rice bran oil was considered to yield 30 kg of rice bran wax. This relationship was used to scale the environmental burdens associated with rice bran oil production to the amount of rice bran wax required in the coating formulation. For the functional unit, a total of 0.045 kg of rice bran wax was assumed. This approach assumes that the environmental burdens associated with rice bran oil production are proportionally attributed to the wax fraction based on the reported wax yield.

Impact Category	Rice bran oil production (1000 kg oil)	Equivalent rice bran wax output (30 kg wax)	Rice bran wax required for FU (0.045 kg wax)
ADP elements (kg Sb eq.)	3.82×10^{-4}	1.11×10^{-5}	1.67×10^{-8}
ADP fossil (MJ)	2.09×10^{4}	6.09×10^{2}	9.14×10^{-1}
AP (kg SO2 eq.)	2.40×10^{1}	6.99×10^{-1}	1.05×10^{-3}
EP (kg Phosphate eq.)	4.24×10^{0}	1.24×10^{-1}	1.85×10^{-4}
FAETP (kg DCB eq.)	2.96×10^{0}	8.61×10^{-2}	1.29×10^{-4}
GWP (kg CO2 eq.)	2.11×10^{3}	6.16×10^{1}	9.24×10^{-2}
HTP (kg DCB eq.)	3.65×10^{1}	1.06×10^{0}	1.60×10^{-3}
MAETP (kg DCB eq.)	2.51×10^{4}	7.30×10^{2}	1.09×10^{0}
ODP (kg R11 eq.)	0	0	0
POCP (kg Ethene eq.)	1.76×10^{0}	5.12×10^{-2}	7.67×10^{-5}
TETP (kg DCB eq.)	3.81×10^{-1}	1.11×10^{-2}	1.66×10^{-5}

Table S4. Estimated environmental impacts of rice bran wax production based on a 3% wax yield from rice bran oil production.

Section 3.2 – Economic allocation approach (sensitivity analysis)

Market prices and product value shares for rice bran oil processing co-products were obtained from Kabir et al. (24) for a rice bran oil mill in Bangladesh (2011). These data were used to estimate the economic allocation factor for rice bran wax.

Product	Market price ($/kg)	Product value (%)
Rice bran oil (aggregated packed + loose)	1.34	53%
De-oiled rice bran	0.20	40%
Fatty acid	0.78	6%
Rice bran wax	0.43	1%
Gum	0.07	<1%
Splint earth	0.03	<1%

Table S5. Market prices and value shares of rice bran oil processing co-products.

Rice bran wax represents approximately 1% of the total economic value of refinery outputs. Therefore, 1% of the total environmental burdens of rice bran oil production (1000 kg system) were allocated to wax. Assuming a 3% wax yield relative to oil mass, impacts were normalized to 1 kg rice bran wax and then scaled to the functional unit (0.045 kg).

Impact Category	Rice bran oil production (1000 kg oil)	Allocated to wax (1%, economic)	Rice bran wax required for FU (0.045 kg wax)
ADP elements (kg Sb eq.)	3.82×10^{-4}	3.82×10^{-6}	5.73×10^{-9}
ADP fossil (MJ)	2.09×10^{4}	2.09×10^{2}	3.14×10^{-1}
AP (kg SO2 eq.)	2.40×10^{1}	2.40×10^{-1}	3.60×10^{-4}
EP (kg Phosphate eq.)	4.24×10^{0}	4.24×10^{-2}	6.36×10^{-5}
FAETP (kg DCB eq.)	2.96×10^{0}	2.96×10^{-2}	4.44×10^{-5}
GWP (kg CO2 eq.)	2.11×10^{3}	2.11×10^{1}	3.17×10^{-2}
HTP (kg DCB eq.)	3.65×10^{1}	3.65×10^{-1}	5.48×10^{-4}
MAETP (kg DCB eq.)	2.51×10^{4}	2.51×10^{2}	3.76×10^{-1}
ODP (kg R11 eq.)	0	0	0
POCP (kg Ethene eq.)	1.76×10^{0}	1.76×10^{-2}	2.63×10^{-5}
TETP (kg DCB eq.)	3.81×10^{-1}	3.81×10^{-3}	5.71×10^{-6}

Table S6. Environmental impacts allocated to rice bran wax using economic allocation.

Section 4 – Scaling assumptions

The life cycle inventory was constructed using the experimental reference flow corresponding to the coating of a single substrate with an area of 0.002 m². Inventory flows were scaled to the functional unit defined in the main manuscript by proportional extrapolation of the experimentally measured material and energy inputs. Transportation impacts were calculated using tonne-kilometre (tkm) units based on the mass of the transported material and the estimated transport distance. Scaling from the experimental reference flow to the functional unit was performed assuming linear proportionality between coated surface area and material and energy consumption.

Stage	Flow type	Sub-stage	Value (experiment)	Value (Functional Unit)	Unit
Raw materials					
	Input	N-hexane	0.006	3.0	L
	Input	Rice bran wax	9.0×10^{-5}	0.045	Kg
Transportation					

		N-hexane to lab			
	Transportation	Leg A (US inland)	2.00×10^{-4}	8.10×10^{-2}	tkm
	Transportation	Leg B (ocean)	2.05×10^{-2}	1.02×10^{1}	tkm
	Transportation	Leg C (UK inland)	2.00×10^{-4}	8.00×10^{-2}	tkm
		Rice bran wax to lab			
	Transportation	Leg D (UK inland)	1.00×10^{-5}	6.00×10^{-3}	tkm
Dispersion preparation					
	Utility	Mechanical stirring (electricity)	0.007	3.5	kWh
	Output	Coating dispersion	0.004	2.1	Kg

Table S7. Scaling of experimental inputs to the functional unit.

Section 5 – Datasets

The background processes used in the life cycle assessment model were sourced primarily from the Ecoinvent 3.11 database as implemented in LCA for Experts (GaBi) 10.9.3.0. **Table S8** summarizes the datasets used to represent the main processes included within the system boundary.

**Process**	**Dataset used**	**Source**
N-hexane production	RER: hydrocarbons purification, molecular sieve separation of naphtha	Ecoinvent 3.11
Petroleum ether	RER: naphtha production, petroleum refinery operation	Ecoinvent 3.11
Rice bran wax production (mass allocation)	Derived from rice bran oil LCA data using wax yield	Literature (20)
Rice bran wax production (economic allocation)	Derived from rice bran oil data using economic allocation factors	Literature (24)
Electricity supply (baseline)	GB: market for electricity, low voltage	Ecoinvent 3.11
Electricity supply (renewable scenario)	CH: market for electricity, low voltage, renewable energy products	Ecoinvent 3.11
Road transport (US)	BR: transport, freight, lorry, 16-32 metric ton, diesel, unregulated	Ecoinvent 3.11
Road transport (UK)	RER: transport, freight, lorry, 16-32 metric ton, diesel, EURO 6	Ecoinvent 3.11
Ocean transport (US-UK)	GLO: transport, freight, sea, container ship, heavy fuel oil	Ecoinvent 3.11

Table S8. Background datasets used to represent the main processes included in the life cycle inventory model.
